# Mechanistic Insights into the Role of C-Type Lectin Receptor/CARD9 Signaling in Human Antifungal Immunity

**DOI:** 10.3389/fcimb.2016.00039

**Published:** 2016-04-05

**Authors:** Rebecca A. Drummond, Michail S. Lionakis

**Affiliations:** Fungal Pathogenesis Unit, Laboratory of Clinical Infectious Diseases, National Institute of Allergy and Infectious Diseases, National Institutes of HealthBethesda, MD, USA

**Keywords:** CARD9, lectins, fungi, Candida, neutrophils, innate immunity

## Abstract

Human CARD9 deficiency is an autosomal recessive primary immunodeficiency disorder caused by biallelic mutations in the gene *CARD9*, which encodes a signaling protein that is found downstream of many C-type lectin receptors (CLRs). CLRs encompass a large family of innate recognition receptors, expressed predominantly by myeloid and epithelial cells, which bind fungal carbohydrates and initiate antifungal immune responses. Accordingly, human CARD9 deficiency is associated with the spontaneous development of persistent and severe fungal infections that primarily localize to the skin and subcutaneous tissue, mucosal surfaces and/or central nervous system (CNS). In the last 3 years, more than 15 missense and nonsense *CARD9* mutations have been reported which associate with the development of a wide spectrum of fungal infections caused by a variety of fungal organisms. The mechanisms by which CARD9 provides organ-specific protection against these fungal infections are now emerging. In this review, we summarize recent immunological and clinical advances that have provided significant mechanistic insights into the pathogenesis of human CARD9 deficiency. We also discuss how genetic mutations in CARD9-coupled receptors (Dectin-1, Dectin-2) and CARD9-binding partners (MALT1, BCL10) affect human antifungal immunity relative to CARD9 deficiency, and we highlight major understudied research questions which merit future investigation.

The last 3 years have seen an increasing number of reports of patients with overwhelming fungal disease attributed to a genetic mutation in the gene *CARD9* (Caspase Recruitment Domain Family, member 9). Human CARD9 deficiency was first described in 2009 in a consanguineous family afflicted with mucosal and lethal systemic infections caused by *Candida* species, (Glocker et al., 2009), which are common human commensal yeasts (Lionakis and Netea, 2013). Since then, more than 15 other *CARD9* mutations have been identified in 46 patients, that uniquely associate with mucosal and/or systemic infections caused by specific fungal phyla (Drewniak et al., 2013; Lanternier et al., 2013; Gavino et al., 2014; Lanternier et al., 2014; Wang et al., 2014; Drummond et al., 2015; Gavino et al., 2015; Grumach et al., 2015; Herbst et al., 2015; Jachiet et al., 2015; Lanternier et al., 2015; Liang et al., 2015; Celmeli et al., 2016; Yan et al., 2016). These reports unequivocally demonstrate the critical dependence on CARD9 in human antifungal immunity.

Fungi are recognized by the innate immune system via pattern recognition receptors (PRRs) belonging to several different classes, including the C-type lectin receptors (CLRs), Toll-like receptors (TLRs), and the intracellular nucleotide-binding oligomerization domain-like receptors (NLRs), which are predominantly expressed by myeloid cells including neutrophils, monocytes, macrophages, and dendritic cells (Hardison and Brown, 2012). Although all of these receptor families are involved in innate antifungal recognition, only mutations in the CLR pathway have thus far been associated with the spontaneous development of fungal infections in humans.

Many members of the CLR superfamily bind fungal carbohydrates and initiate complex intracellular signaling pathways to mediate cellular functions, such as fungal killing and production of pro-inflammatory cytokines (Lionakis and Netea, 2013). Several CLRs utilize a common signaling pathway involving the kinase Syk and the signaling adaptor, CARD9 (Gross et al., 2006; Drummond et al., 2011). Thus, CARD9 is centrally positioned downstream of many CLRs and is critical for their function.

In this review, we highlight recent novel immunological insights into the pathogenesis of human CARD9 deficiency and summarize the different human *CARD9* mutations described to date along with their clinical and functional characteristics (Table 1). In addition, we discuss how genetic mutations in the CARD9-coupled receptors (e.g., Dectin-1, Dectin-2) and molecules that form signaling complexes with CARD9 (e.g., MALT1, BCL10) affect human antifungal immunity (Table 2), while highlighting major understudied areas that merit future investigation.

**Table 1:** **Summary of human *CARD9* mutations reported to date, with their associated clinical, immunological and functional characteristics**.

**Mutation**	**Location**	**Associated fungal disease(s)**	**Phagocyte functional defects**	Cytokine defects^*^	Age of onset in years (range)^**^	**Peripheral blood Th17 cells**	**Reference(s)**
**MISSENSE**							
p.R57H (homozygous)	CARD domain	Mucosal; CMC Systemic; *Candida albicans* meningoencephalitis, vertebral osteomyeltis	Neutrophil killing (unopsonized yeast) Monocyte killing CNS neutropenia	TNFα, IL-6, IL17A/F, IFNγ, IL-1β, IL12p70	1 (mucosal); 8 (systemic)	Normal	Drummond et al., 2015
p.R18W (homozygous)	CARD domain	Systemic; *Exophiala dermatitidis* brain and liver infection	NR	IL-6, TNFα	5	Normal^a^	Lanternier et al., 2014
p.R101C (homozygous)	CARD domain	Systemic; *Trichophyton rubrum* and *Trichophyton violaceum* deep dermatophytosis^***^	NR	IL-6	NR	Low	Lanternier et al., 2013
p.R101L (homozygous)	CARD domain	Mucosal; CMC Systemic; *Trichophyton mentagrophytes* deep dermatophytosis^***^	Neutrophil killing	NR	3 (mucosal); 11 (systemic)	NR	Grumach et al., 2015
p.Y91H (homozygous or compound heterozygous with c.-529T>C)	CARD domain	Systemic; *Candida albicans* meningoencephalitis, vertebral osteomyelitis	NR	GM-CSF	30, 38, 39	Normal^a^	Gavino et al., 2014, 2015
p.R373P (compound heterozygous with p.G72S)	Coiled-coil domain	Systemic; *Candida dubliensis* meningoencephalitis	Neutrophil killing (unopsonized yeast) CNS neutropenia	IL-6, IL-1β, IL-8	7	Low^a^	Drewniak et al., 2013
p.G72S	CARD domain	Systemic; *Candida dubliensis* meningoencephalitis	Neutrophil killing (unopsonized yeast) CNS neutropenia	IL-6, IL-1β, IL-8	7	Low^a^	Drewniak et al., 2013
p.R35Q (homozygous)	CARD domain	Mucosal; *Candida glabrata*-induced colitis Systemic; *Candida glabrata* meningoencephalitis and sinus disease	NR	IL-6, TNFα	17	NR	Lanternier et al., 2015
p.R70W (homozygous)	CARD domain	Mucosal; CMC Systemic; *Candida albicans* meningoencephalitis	CNS neutropenia	IL-6, TNFα	7, 39	Normal	Lanternier et al., 2015
**NONSENSE**							
p.Q289X (homozygous)	Coiled-coil domain	Mucosal; CMC Systemic; *T. rubrum* and *T. violaceum* deep dermatophytosis^***^, *Candida albicans* meningoencephalitis	NR	lL-6, TNFα	5–21, 37, 13	Low	Lanternier et al., 2013, 2015; Jachiet et al., 2015
p.L64fsX59 (homozygous or compound heterozygous with p.Q158X)	CARD domain	Systemic; *Phialophora verrucosa* phaeohyphomycosis, *Corynespora cassiicola* phaeohyphomycosis	Neutrophil killing (unopsonized yeast)	IL-6, TNFα, IL-1β, IL-23p19	13, 35	Low	Wang et al., 2014; Liang et al., 2015; Yan et al., 2016
p.Q158X	Coiled-coil domain	Mucosal; *Phialophora verrucosa* phaeohyphomycosis	Neutrophil killing (unopsonized yeast)	IL-6, TNFα, IL-1β, IL-23p19	13	Low	Wang et al., 2014; Liang et al., 2015;
p.D274fsX60 (homozygous)	Coiled-coil domain	Mucosal; *Phialophora verrucosa* phaeohyphomycosis	Neutrophil killing (unopsonized yeast)	IL-6, TNFα, IL-1β, IL-23p19	6, 20, 48	Low	Wang et al., 2014; Liang et al., 2015;
p.E232del (homozygous)	Coiled-coil domain	Systemic; *Exophiala* spinifera bone, lung and subcutaneous tissue infection	NR	NR	18	NR	Lanternier et al., 2014
p.Q295X (homozygous)	Coiled-coil domain	Mucosal; CMC, VVC, superficial dermatophytosis, *Candida albicans*-induced colitis Systemic; *Candida albicans* meningoencephalitis	Neutrophil killing (unopsonized yeast) CNS neutropenia	IL-6, TNFα, IL-1β	3-42, 26, 4, 3	Normal, Lanternier et al., 2015; Herbst et al., 2015 Low, Glocker et al., 2009; Celmeli et al., 2016	Glocker et al., 2009; Herbst et al., 2015; Lanternier et al., 2015; Celmeli et al., 2016
c.-529T>C^b^	Promoter	Systemic; *Candida albicans* meningoencephalitis, vertebral osteomyelitis	NR	GM-CSF	30, 38, 39	Normal^a^	Gavino et al., 2015

* Cytokine production assessed by assays using purified myeloid cells or PBMCs stimulated with a fungal stimulus (e.g. heat-killed Candida albicans, curdlan, zymosan) and analyzed by an ELISA-based system.

** Age of onset is given, where available, and relates to the age at which symptoms first developed. Ranges are given for studies analysing more than 1 patients, otherwise ages are given for individual patients.

*** Significant proportion of patients with deep dermatophytosis had involvement of lymph node, whereas a small proportion had bone or brain involvement).

a This was assessed by measuring IL-17 production from restimulated T-cells or whole blood. All other studies used FACS to enumerate IL-17^+^CD4^+^ T-cells in the peripheral blood.

b This mutation results in biallelic imbalance, and only leads to disease in patients who are heterozygous for a second CARD9 mutation, namely p.Y91H in French-Canadian patients. NR, not reported; CMC, chronic mucocutaneous candidiasis; VVC, vulvovaginal candidiasis.

**Table 2:** **Summary of infection susceptibilities to fungal and non-fungal pathogens in humans and mice deficient (or expressing non-functional variants) in the indicated CARD9-coupled receptors and associated signaling molecules**.

	**Human**		**Mouse**		**Selected References**
	**Associated fungal infections**	**Susceptibility to non-fungal pathogens**	**Associated fungal infections**	**Susceptibility to non-fungal pathogens**	
**CARD9-COUPLED RECEPTORS**					
Dectin-1	Candidiasis (mucosal, VVC), dermatophytosis	NR	Systemic candidiasis, pulmonary aspergillosis, pulmonary pneumocystis pulmonary coccidioidomycosis	NR	Drummond et al., 2011
Dectin-2	NR	NR	Systemic candidiasis	Streptococcal pneumonia	Akahori et al., 2016
Dectin-3	NR	NR	Systemic candidiasis	Mycobacterial disease	Zhu et al., 2013; Wilson et al., 2015
Mincle	NR	NR	Systemic candidiasis	NR	Hardison and Brown, 2012
**SIGNALING MOLECULES**					
CARD9	See Table 1	NR	Systemic candidiasis, pulmonary aspergillosis	*Listeria monocytogenes*, mycobacterial disease	Gross et al., 2006; Hsu et al., 2007; Dorhoi et al., 2010; Jhingran et al., 2012
BCL10	CMC	Bacterial gastrointestinal infections (*Campylobacter, Clostridium difficile*), viral gastrointestinal and pulmonary infections (adenovirus, influenza)	NR	Viral infections (LCMV, VSV)	Ruland et al., 2001; Torres et al., 2014; de Diego et al., 2015
MALT1	CMC	Bacterial pneumonia, CMV	NR	NR	Jabara et al., 2013; de Diego et al., 2015
Trim62	NR	NR	Systemic candidiasis	NR	Cao et al., 2015
Rubicon	NR	NR	Systemic candidiasis^*^	Influenza A^*^	Yang et al., 2012

* These results were obtained using viral vectors to either increase or decrease Rubicon expression in vivo. Rubicon expression adversely affected immunity to these pathogens, whereas viruses carrying silencing RNA enhanced protection. NR, not reported; CMC, chronic mucocutaneous candidiasis; VVC, vulvovaginal candidiasis; LCMV, lymphocytic choriomeningitis virus; VSV, vesicular stomatitis virus; CMV, cytomegalovirus.

## Human CARD9 deficiency uniquely predisposes to mucosal and systemic fungal diseases

The vast majority of inborn errors in immunity that predispose to fungal disease typically also lead to the development of non-fungal infections. Furthermore, these disorders usually segregate cleanly between mucosal or systemic fungal disease susceptibility, whereas CARD9 deficiency predisposes to both mucosal and systemic fungal disease. The only other inherited conditions that match the combined susceptibility to mucosal and systemic fungal infections seen in CARD9 deficiency are gain-of-function *STAT1* mutations and autosomal-dominant *STAT3* mutations; however, these mutations also result in bacterial, mycobacterial and/or viral infections (reviewed in detail here Lionakis et al., 2014). In contrast, *CARD9* mutations in humans have thus far only been associated with fungal infections (Table 2), although *Card9*^−∕−^ mice have been reported to be susceptible to intracellular bacterial infections (Hsu et al., 2007; Dorhoi et al., 2010) in addition to their susceptibility to fungal disease.

In mouse models of fungal challenge, Card9 genetic deletion abolishes the ability to control systemic *Candida albicans* infections, with 100% mortality observed as early as 72 h post-infection (Gross et al., 2006; Drummond et al., 2015). Similar results were recently obtained with *Candida tropicalis*, an emerging non-*albicans Candida* species, where fungal growth was found to be uncontrolled in the kidney, spleen, liver, and brain (Whibley et al., 2015). *Card9*^−∕−^ animals were also found to be mildly susceptible to mucosal candidiasis; however, this was only evident during secondary infection suggesting that the dependence on Card9 lies within the adaptive immune compartment for this particular disease (Bishu et al., 2014). The difference between human and mouse susceptibility to mucosal fungal disease in the setting of CARD9-deficiency may be a reflection of the fact that, as opposed to humans, *Candida* is not a commensal organism in mice (Table 2). Susceptibility to pulmonary disease caused by the inhaled mold *Aspergillus fumigatus* is modestly enhanced in *Card9*^−∕−^ animals, but only when these animals are challenged with an overwhelming number of conidia (8 × 10^7^; Jhingran et al., 2012), whereas challenge with the still supra-physiologic inoculum, 3 × 10^7^ conidia, does not confer heightened susceptibility in *Card9*^−∕−^ mice (Jhingran et al., 2015). These mouse data are in line with the lack of an association between pulmonary mold infections and human CARD9-deficiency to date. More work will be required in mice to determine the role of Card9 in host defense against non-*Candida*, non-*Aspergillus* fungal pathogens that have been shown to cause disease in CARD9-deficient patients (Table 1).

Sixteen human *CARD9* mutations have now been identified and characterized in patients originating from the USA, Canada, Europe, Middle East, South America, and China (Glocker et al., 2009; Drewniak et al., 2013; Lanternier et al., 2013; Gavino et al., 2014; Lanternier et al., 2014; Wang et al., 2014; Drummond et al., 2015; Grumach et al., 2015; Herbst et al., 2015; Jachiet et al., 2015; Lanternier et al., 2015; Yan et al., 2016). These mutations are found in the promoter region and the coiled-coil and CARD protein domains of CARD9, and both nonsense and missense mutations have been described (Table 1). Consistent with an autosomal recessive mode of inheritance, 80% of the reported patients were from consanguineous families; 89% were homozygous for the same allele, whereas 11% were compound heterozygous for different alleles. These mutations have variable penetrance, age of onset and effects, such that CARD9-deficiency manifests with multiple diseases caused by a variety of fungal organisms (Table 1). Based on the observed clinical variability thus far, the manifestations of human CARD9 deficiency can be broadly divided into three disease syndromes.

The first syndrome associated with CARD9 deficiency affects the skin and subcutaneous tissues, and patients in this category present with dramatic persistent fungal infections of the skin, nails and/or scalp (Lanternier et al., 2013; Wang et al., 2014), with occasional contiguous dissemination to the subcutaneous layers, lymph nodes and bone (Lanternier et al., 2013). The etiological agents for these infections so far described include *Trichophyton* species (causing deep dermatophytosis; Lanternier et al., 2013), *Phialophora verrucosa* (an agent of phaeohyphomycosis; Wang et al., 2014), and one intriguing case of phaeohyphomycosis caused by the plant pathogen *Corynespora cassiicola* (Yan et al., 2016). The CARD9-dependent functions that provide organ-specific protection in the skin against dermatophytes and agents of phaehyphomycosis are currently not well defined. In mouse models of cutaneous *C. albicans* infection, it has been shown that IL-17A and IL-23 signaling are critical for antifungal host defense in the skin (Kagami et al., 2010), and that induction of Th17 immunity is dependent on the CARD9-coupled receptor Dectin-1 expressed by Langerhans cells (Kashem et al., 2015), a population of dendritic cells resident in the epidermal layers of the skin. Both of these studies used *C. albicans* to initiate infection and did not investigate the dependence on Card9 for these activities. It therefore remains to be explored whether IL-17-dependent mechanisms of clearance require CARD9 for antifungal defense in the skin, and how important these mechanisms are for clearance of dermatophytes and agents of phaeohyphomycosis, where innate killing mechanisms of neutrophils and macrophages appear important (de Sousa et al., 2015; Liang et al., 2015).

The second predominant manifestation of human CARD9-deficiency is systemic fungal disease, which primarily manifests as fungal meningoencephalitis caused by *Candida* species (Gavino et al., 2014; Drummond et al., 2015). Some of these patients have also developed accompanying bone infection of the vertebra (Gavino et al., 2014; Drummond et al., 2015). Less often, CARD9-deficient patients develop phaeohyphomycosis caused by *Exophiala* species that targets the central nervous system (CNS) and/or other deep tissues including the liver, lung, and bone (Lanternier et al., 2014). Strikingly, *Candida* infections involving the kidney, liver or spleen, the organs typically affected in patients with iatrogenic immunosuppression (Lionakis, 2014), have not been reported in CARD9-deficient patients. Similarly, pulmonary infections by ubiquitous inhaled molds, or infections by *Cryptococcus neoformans, Cryptococcus gattii, Pneumocystis jirovecii*, and the endemic dimorphic fungi (i.e., *Histoplasma capsulatum, Coccidioides immitis*, or *Blastomyces dermatitidis*) have also not been reported thus far in CARD9-deficient patients. Collectively, the tropism of organ-specific systemic fungal disease in CARD9 deficiency implies that CARD9 has fungal- and organ-specific innate immune effects, which we currently only partially understand.

In addition to cutaneous/subcutaneous and CNS fungal disease, many CARD9-deficient patients are also susceptible to persistent and recurrent infections of the mucosal surfaces by *Candida* species, collectively termed chronic mucocutaneous candidiasis (CMC). CMC can develop as a single entity or in association with the aforementioned systemic or cutaneous/subcutaneous fungal diseases, and the mouth, gut and vagina have all been reportedly affected in CARD9-deficient patients (Table 1). When CMC and systemic fungal disease occur in the same individual, it appears that CMC starts at an earlier age and often pre-dating the development of the more serious fungal diseases affecting the brain (Drummond et al., 2015; Grumach et al., 2015; Lanternier et al., 2015). CARD9 deficiency is the only reported human genetic disorder where mucosal and systemic *Candida* disease occur together in the absence of other non-fungal infections (Lionakis et al., 2014). This is a rare event for patients to develop both mucosal and systemic *Candida* infections, since each disease has differing requirements for immune protection. Specifically, mucosal candidiasis control requires IL-17 signaling via Th17 cells, γδ T-cells and innate lymphoid cell populations (Conti et al., 2009; Gladiator et al., 2013; Conti et al., 2014), whereas systemic defense depends heavily on the accumulation and effector function of neutrophils and mononuclear phagocytes (Brown, 2011; Lionakis and Netea, 2013; Lionakis, 2014). Therefore, CARD9 deficiency gives rise to detrimental effects in both lymphoid and myeloid cell accumulation and/or function.

## Impaired induction of IL-17 responses may account for mucosal fungal disease in CARD9-deficient humans

As CARD9-deficiency is associated with a spectrum of fungal diseases, this could indicate at the varying requirements for CARD9-dependent functions at different anatomical sites, since antifungal immune responses vary drastically between organs (Lionakis et al., 2011). As mentioned above, Th17 responses are required for protection against mucosal candidiasis in both mice and humans, whereas there is far less dependence on these responses for systemic anti-*Candida* defense (Conti and Gaffen, 2015). The critical role of CARD9 in the induction of IL-17 responses (LeibundGut-Landmann et al., 2007; Bishu et al., 2014) may account for CMC susceptibility of affected patients. Indeed, the majority of *CARD9* mutations are associated with a reduced number of circulating Th17 cells (Table 1). However, approximately half of the reported *CARD9* missense mutations have no impact on the percent of peripheral blood Th17 cells following stimulation with PMA/ionomycin (Table 1). Thus, the role of CARD9 in human Th17 immunity and defense against CMC still remains unclear. Further work will be needed to decipher how human CARD9 deficiency affects the pathways governing Th17 differentiation and function, and how *CARD9* mutations that do not affect peripheral blood Th17 cell numbers can still result in the development of CMC.

## CARD9 is required for neutrophil recruitment to the fungal-infected CNS

Our group's research recently uncovered novel insight into how human CARD9-deficiency may predispose to fungal infection of the CNS (Drummond et al., 2015). Prior to this work, the susceptibility to CNS fungal disease was thought to be the result of a selective defect in killing unopsonized *C. albicans* yeasts by CARD9-deficient neutrophils (Drewniak et al., 2013; Drummond et al., 2015). Since opsonization is naturally low in the CNS, this killing defect has been thought to be clinically relevant in this tissue thus potentially accounting for localized organ-specific disease. Neutrophils are critical for antifungal host defense in mice and humans (Lionakis and Netea, 2013), and an intrinsic functional defect in these cells is a plausible explanation for the susceptibility to fungal CNS disease in CARD9-deficient patients. However, *C. albicans* pseudohyphae, not yeast, is the predominant fungal morphology observed in infected CNS tissue of mice and humans (Lionakis et al., 2011; Drummond et al., 2015), and hyphal killing by CARD9-deficient human neutrophils appears intact (Drewniak et al., 2013; Drummond et al., 2015). Importantly, we found that neutrophils are not recruited to the fungal-infected CNS in a CARD9-deficient patient or infected *Card9*^−∕−^ animals (Drummond et al., 2015). In line with our findings, other groups have also reported a lack of neutrophil accumulation in the *Candida*-infected CSF of CARD9-deficient patients; instead, large numbers of lymphocytes and eosinophils comprise the cellular inflammatory reaction in these patients (Drewniak et al., 2013; Lanternier et al., 2015; Celmeli et al., 2016). This is in stark contrast to patients with *Candida* meningoencephalitis who have intact CARD9 signaling, where cells recruited to the infected CSF are predominantly neutrophils (Drummond et al., 2015). Therefore, although the selective neutrophil killing defect against unopsonized *Candida* yeasts may contribute toward the development of CNS fungal disease in CARD9-deficient patients, the major contributor toward the CNS infection susceptibility of CARD9-deficient patients appears to be the lack of neutrophils in the infected CNS, thus creating a condition of “CNS-specific neutropenia.”

We investigated the underlying mechanisms for the CNS-specific neutropenia observed in our CARD9-deficient patient and *Card9*^−∕−^ animals. Impaired neutrophil accumulation in the *Candida*-infected CNS was not the result of defective neutrophil production in the bone marrow, neutrophil egress into the blood, neutrophil survival or neutrophil-intrinsic chemotaxis (Drummond et al., 2015). Instead, we found that CARD9 deficiency confers a lack of CXC-chemokine induction in the CNS during fungal infection, in both mice and humans (Drummond et al., 2015). Using mouse models, we found that CXC-chemokines were induced in a proportional manner to brain fungal burden, and this relationship was lost in the absence of Card9 (Drummond et al., 2015). We showed that this defect is specific to the CNS, since CXC-chemokine production and neutrophil recruitment were affected in a significantly lesser extent in the kidney of infected *Card9*^−∕−^ mice, similar to recent findings using *C. tropicalis* (Whibley et al., 2015). Furthermore, we also showed that this defect was specific to fungal infection, as neutrophil recruitment was also unaffected following bacterial brain infection (Drummond et al., 2015), in agreement to the lack of susceptibility to bacterial meningoencephalitis in CARD9-deficient patients. Future work will be required to define the Card9-dependent cellular sources of neutrophil-targeted CXC-chemokines that are required for neutrophil recruitment to the CNS during fungal invasion, as well as the CARD9-coupled receptors critical for this effector cell accumulation. Of interest, Dectin-1 has been shown to promote neutrophil recruitment in the *C. albicans*-infected mouse peritoneum (Taylor et al., 2007), but its role in neutrophil recruitment in the fungal-infected CNS has not yet been explored.

Consistent with a role of CARD9 in neutrophil accumulation during fungal disease, the Hohl group recently characterized a neutrophil recruitment defect to the *A. fumigatus*-infected lung in *Card9*^−∕−^ mice, which was also linked to poor CXC-chemokine production in the lung (Jhingran et al., 2015). However, in that model, Card9 was only partially required in the later stages of infection for neutrophil accumulation in the infected lung. Instead, neutrophil recruitment in the early phase was controlled by the TLR/IL-1R signaling adaptor, MyD88, which was required for chemokine induction by epithelial cells (Jhingran et al., 2015). Thus, the collective dependence on Card9 for neutrophil recruitment during pulmonary mold infections appears relatively mild compared to the universal reliance required for neutrophil accumulation and protection against *Candida* infection in the CNS (Gross et al., 2006; Drummond et al., 2015; Whibley et al., 2015). This dichotomy observed in mice between the Card9-dependence for protection against molds and yeasts may help explain why human CARD9 deficiency does not appear to associate with pulmonary fungal infections, despite the continuous environmental exposure to fungal spores. In order to clarify the fungal- and organ-specific functions of CARD9, future studies will be needed to examine the mechanistic role of CARD9 on other myeloid cells, besides neutrophils, such as monocytes, resident macrophages, dendritic cells, and myeloid-derived suppressor cells (Rieber et al., 2015), as well as resident epithelial and stromal cells in mediating systemic antifungal host defense.

## Immune-based adjunct treatments may improve the outcome of CARD9-deficient patients with *Candida* meningoencephalitis

Immune-based strategies have recently been reported to be an effective adjunct treatment for fungal meningoencephalitis in CARD9-deficient patients (Gavino et al., 2014, 2015; Celmeli et al., 2016), as long-term antifungal usage does not always lead to clinical remission, especially when treatment is not aggressive early on (Drummond et al., 2015). For instance, Gavino et al. showed that exogenous treatment with granulocyte-macrophage colony stimulating factor (GM-CSF) successfully led to clinical remission in a cohort of French-Canadian patients with *Candida* CNS infection, carrying the p.Y91H allele (Gavino et al., 2014, 2015). More recently, this work was mirrored by a second group using the related colony stimulating factor, G-CSF, administration of which resulted in clinical improvement in a Turkish patient with the p.Q295X mutation who had uncontrolled *Candida* CNS infection (Celmeli et al., 2016). Long-term GM-CSF treatment appeared necessary in the French-Canadian patients as its discontinuation led to clinical relapse, whereas short-term G-CSF treatment appeared adequate as its discontinuation did not result in infection relapse (Gavino et al., 2014, 2015; Celmeli et al., 2016).

At the mechanistic level, the Y91H mutation was shown to cause a defect in the formation of a CARD9-Ras protein-specific guanine nucleotide releasing factor 1 (Ras-GRF1) complex, necessary for downstream NF-κB and ERK signaling, which subsequently led to defective GM-CSF production in response to fungal ligands (Gavino et al., 2015). Impaired ERK activation via altered Card9 association with Ras-GRF1 and H-Ras was confirmed in *Card9*^−∕−^ mice (Jia et al., 2014). Presumably, this GM-CSF specific defect may be amenable to correction by exogenous GM-CSF administration in these patients. In the study by the Grimbacher group, G-CSF treatment was shown to correct defective IL-17 production from CARD9-deficient cells, although not to the same levels as CARD9^+^ cells donated by a healthy volunteer (Celmeli et al., 2016). Both GM-CSF and G-CSF have pleiotropic effects on the immune system, and could therefore have potentially rescued CNS infection via boosting neutrophil recruitment to the site of infection and/or enhancing myeloid cell effector function, or other as yet unknown mechanisms may be involved. As these parameters were not examined in these studies, it is unclear whether GM-CSF/G-CSF could bypass multiple immune defects associated with human CARD9 deficiency, or if these success stories rely on correcting specific defects caused by the *CARD9* mutation identified in these particular patients. Additional work testing the efficacy of GM-CSF/G-CSF in other CARD9-deficient patients with different mutations and other clinical manifestations beyond *Candida* CNS infection, corroborated by similar studies in *Card9*^−∕−^ mouse models of fungal infections, will be required to determine the general applicability and the accompanying mechanisms of these treatments in human CARD9-deficiency.

## CLR mutations do not result in development of systemic fungal infections in humans

After ligand binding, CLRs initiate intracellular signaling either directly via their intracellular tails (e.g., Dectin-1) or indirectly via association with a signaling adaptor such as FcRγ (e.g., Dectin-2, Mincle; Drummond et al., 2011). In the Syk/CARD9 pathway, tyrosine residues in the signaling motif of the CLR tail or adaptor become phosphorylated by SHP-2 leading to recruitment and activation of Syk kinase (Deng et al., 2015). Downstream of Syk, CARD9 forms a signaling complex with BCL10 and MALT1 which provides a scaffold for further signaling events such as NF-κB activation and the formation and activation of inflammasomes (Lionakis and Netea, 2013). The formation and regulation of the CARD9-BCL10-MALT1 (CBM) signaling complex is only partially understood, however recent reports have identified CARD9 binding partners that inhibit or activate CLR-CARD9 signaling. For example, TRIM62 is a ubiquitin ligase that activates CARD9 and thus positively regulates the CLR-CARD9 pathway (Cao et al., 2015). Accordingly, *Trim62*^−∕−^ mice were recently found to be more susceptible to systemic candidiasis, with increased tissue fungal burdens and accelerated mortality (Cao et al., 2015), although this increase was modest relative to that seen in *Card9*^−∕−^ animals that develop overwhelming rapidly-lethal disease (Gross et al., 2006; Drummond et al., 2015). In contrast, the protein Rubicon was shown to be a negative regulator of CARD9 signaling, acting to dissemble the CBM complex and interrupt downstream cytokine production (Yang et al., 2012). For both of these molecules, as well as other yet unknown CARD9 signaling partners, their role in human antifungal immunity remains to be determined (Table 2).

Despite sharing the CARD9 intracellular signaling pathway, many CLRs exhibit independent functions (reviewed extensively elsewhere Hardison and Brown, 2012) and form complex cross-talk relationships that can either be synergistic (Hadebe et al., 2015) or modulatory (Gringhuis et al., 2011). Although individual CLRs perform many critical functions, mutations that disrupt their expression do not lead to development of spontaneous invasive fungal disease, but are instead associated with mucosal infections (Table 2). For example, the best-studied human CLR mutation is p.Y238X in the *CLEC7A* gene, which encodes Dectin-1. Dectin-1 is the β-glucan receptor (Brown, 2006), predominantly expressed by myeloid cells, and is required for many antifungal activities including fungal killing, phagocytosis, cytokine production, inflammasome activation, and CD4^+^ T-cell survival and function (Drummond and Brown, 2011; Gringhuis et al., 2012; Drummond et al., 2016). The Y238X polymorphism causes poor expression of Dectin-1 and defective pro-inflammatory cytokine production following fungal restimulation (Ferwerda et al., 2009). A family of patients with this mutation was reported to suffer from frequent bouts of vulvovaginal candidiasis and onychomycosis (Ferwerda et al., 2009). In line with this, IL-17 responses were found to be defective in Y238X cells restimulated with *C. albicans* yeasts (van de Veerdonk et al., 2009), and thus disruption of this protective response could be responsible for the association between Y238X and mucosal fungal infections described in the above family. However, the precise role of the Y238X polymorphism in promoting mucosal fungal disease deserves further investigation, since this polymorphism has a high prevalence in several human populations (Ferwerda et al., 2009), yet it is unclear to what extent carriers of Y238X suffer from such infections. At the population level, patients carrying the Y238X polymorphism were shown to be more likely to have oral and gastrointestinal *Candida* colonization (Plantinga et al., 2009), and were at greater risk for developing invasive mold infections following hematopoietic stem cell transplantation (Cunha et al., 2010), at odds with the absence of pulmonary fungal infections in CARD9-deficient patients. At the mechanistic level, the Y238X polymorphism has no effect on phagocytosis and fungal killing by neutrophils *in vitro*, which may account for why these patients do not develop systemic disease. However, many *CARD9* mutations also do not affect phagocytosis and have only minor effects on fungal killing (Drewniak et al., 2013; Drummond et al., 2015; Liang et al., 2015), thus there are likely other complex functions dependent on CARD9 and independent of Dectin-1 which we have yet to discover, that are critical for systemic defense.

Other CLR polymorphisms that have been associated with fungal disease in humans include the Dectin-1 polymorphism rs2078178 and the Dectin-2 polymorphism rs11045418. The Dectin-1 variant was found to associate with medically refractory ulcerative colitis, through an as yet unclear mechanism whereby the Dectin-1 variants aggravate existing inflammatory bowel disorders. The authors used mouse models to demonstrate that *C. tropicalis* was responsible for worsening colitis in *Clec7a*^−∕−^ animals, indicative of the involvement of fungi and defective antifungal immunity in this model (Iliev et al., 2012). Dectin-2 is another CLR that binds α-mannans and has a similar expression profile to Dectin-1 (Kerscher et al., 2013), and has been shown to be particularly important for initiation of antifungal Th17 responses (Saijo et al., 2010; Gringhuis et al., 2011). Recently, a Dectin-2 polymorphism was found to associate with pulmonary cryptococcosis, but not with cryptococcal meningitis, in Chinese patients (Hu et al., 2015). The basis for this association remains unclear, since Dectin-2-deficient mice are not more susceptible to pulmonary cryptococcosis (Nakamura et al., 2015) and detailed studies into the functional consequences of human Dectin-2 polymorphisms are currently lacking. Identification of other human mutations in CLRs is still in its infancy (Table 2), as exemplified by the recent characterization of novel CLRs (e.g., Dectin-3, Mincle) and thus the full scope and spectrum of CLR-dependent antifungal immune functions is not yet well defined. More work is therefore required to comprehensively analyze the CLR-specific roles in immune responses to various fungal pathogens.

## Deficiencies in the CARD9 partners MALT1 and BCL10 have broad effects on immunity

While human CARD9 deficiency is uniquely associated with fungal disease, human mutations in its partners *BCL10* and *MALT1* have wider consequences for the immune system (reviewed extensively here de Diego et al., 2015, and see Table 2).

MALT1 is required for NF-κB activation and is found downstream of many immune receptors, including antigen-specific T- and B-cell receptors, the CLRs, and some G-protein coupled receptors (de Diego et al., 2015). Recent reports of patients with *MALT1* missense mutations demonstrated that disruption of MALT1 function has far-reaching consequences for innate and adaptive immunity resulting in combined immunodeficiency. These patients suffered from a variety of infectious and growth disorders, including bacterial pulmonary infections, CMC, persistent cytomegalovirus infection, and inflammatory defects in the gut (Jabara et al., 2013; McKinnon et al., 2014; Punwani et al., 2015). Functionally, MALT1-deficiency conferred an inability to degrade IκBα and defects in T-cell derived IL-2 production (Jabara et al., 2013; McKinnon et al., 2014; Punwani et al., 2015), in line with earlier work describing the immune defects of *Malt1*^−∕−^ mice (de Diego et al., 2015). The effects of human MALT1 deficiency on antifungal immunity have not been studied in detail. However, Gringhuis and colleagues showed that MALT1 was required for production of Th17-polarizing cytokines from human dendritic cells, through a pathway involving Dectin-2 and the NF-κB subunit c-Rel (Gringhuis et al., 2011). Thus, the correlation between human MALT1 deficiency and CMC could be related to the critical role for Th17 cells in protecting mucous membranes from *Candida* infection, although this has yet to be fully explored.

BCL10, like MALT1, is involved in many immune signaling pathways and thus BCL10 deficiency also has broad effects on the hematopoietic and non-hematopoietic immune system. A young child with complete BCL10 deficiency was recently described, who died at 3 years of age following multiple viral infections, CMC, colitis, and gastrointestinal infections (Torres et al., 2014). Interestingly, macrophages and dendritic cells from this patient had normal TLR and Dectin-1 mediated responses, as measured by pro-inflammatory cytokine production following restimulation with specific ligands. Instead, most of the immune defects in this patient mapped to the adaptive immune system, where generation of memory B- and T-cells was severely compromised (Torres et al., 2014), in line with findings using *Bcl10*^−∕−^ animals (Ruland et al., 2001). The study of more patients with *MALT1* and *BCL10* mutations will be required to define the full spectrum of susceptibility to mucosal and systemic fungal disease. In addition, work in mice will be critical to determine the mechanisms by which Malt1 and Bcl10 promote mucosal host defense and whether they mediate CNS-specific protective immunity, as does Card9.

## Concluding remarks

Systemic fungal infections continue to represent a significant unmet medical problem associated with high mortality, making the development of vaccines and novel immune-based treatments highly desirable in order to improve patient outcomes. Deciphering the cellular and molecular immune functions that promote effective antifungal immunity will help us achieve these goals and improve current clinical approaches. Human primary immunodeficiency disorders that predispose to fungal infections give us crucial clues as to which molecules are important for antifungal host defense. CARD9 is a critical antifungal molecule yet there remain significant gaps in our understanding, including the organ-specific functions operating in the skin, mucosal surfaces, and deep tissues, why CARD9-deficiency doesn't predispose to more infections by other fungal organisms, and how some CARD9-deficient patients can live for 2 or 3 decades before developing any serious fungal infections while others with identical mutations develop severe fungal disease early in life. Further study into the pathogenesis of human CARD9-deficiency will yield exciting new insights into the mechanisms that protect humans from these devastating fungal diseases, which should lead to improved therapeutic strategies for affected patients in the future.

## Author contributions

RAD and MSL co-wrote the manuscript and approved for publication.

### Conflict of interest statement

The authors declare that the research was conducted in the absence of any commercial or financial relationships that could be construed as a potential conflict of interest.
